# Pulotu: Database of Austronesian Supernatural Beliefs and Practices

**DOI:** 10.1371/journal.pone.0136783

**Published:** 2015-09-23

**Authors:** Joseph Watts, Oliver Sheehan, Simon J. Greenhill, Stephanie Gomes-Ng, Quentin D. Atkinson, Joseph Bulbulia, Russell D. Gray

**Affiliations:** 1 School of Psychology, University of Auckland, Auckland, New Zealand; 2 Max Planck Institute for the Science of Human History, Jena, Germany; 3 School of Culture, History and Language, ANU College of Asia and the Pacific, Australian National University, Canberra, Australian Capital Territory, Australia; 4 ARC Centre of Excellence for the Dynamics of Language, Australian National University, Canberra, Australian Capital Territory, Australia; 5 School of Art History, Classics and Religious Studies, Victoria University of Wellington, Wellington, New Zealand; 6 Research School of the Social Sciences, Australian National University, Canberra, Australia; 7 Allan Wilson Centre for Molecular Ecology and Evolution, Palmerston North, New Zealand; University of Amsterdam, NETHERLANDS

## Abstract

Scholars have debated naturalistic theories of religion for thousands of years, but only recently have scientists begun to test predictions empirically. Existing databases contain few variables on religion, and are subject to Galton’s Problem because they do not sufficiently account for the non-independence of cultures or systematically differentiate the traditional states of cultures from their contemporary states. Here we present *Pulotu*: the first quantitative cross-cultural database purpose-built to test evolutionary hypotheses of supernatural beliefs and practices. The *Pulotu* database documents the remarkable diversity of the Austronesian family of cultures, which originated in Taiwan, spread west to Madagascar and east to Easter Island–a region covering over half the world’s longitude. The focus of Austronesian beliefs range from localised ancestral spirits to powerful creator gods. A wide range of practices also exist, such as headhunting, elaborate tattooing, and the construction of impressive monuments. *Pulotu* is freely available, currently contains 116 cultures, and has 80 variables describing supernatural beliefs and practices, as well as social and physical environments. One major advantage of *Pulotu* is that it has separate sections on the traditional states of cultures, the post-contact history of cultures, and the contemporary states of cultures. A second major advantage is that cultures are linked to a language-based family tree, enabling the use phylogenetic methods, which can be used to address Galton’s Problem by accounting for common ancestry, to infer deep prehistory, and to model patterns of trait evolution over time. We illustrate the power of phylogenetic methods by performing an ancestral state reconstruction on the *Pulotu* variable “headhunting", finding evidence that headhunting was practiced in proto-Austronesian culture. Quantitative cross-cultural databases explicitly linking cultures to a phylogeny have the potential to revolutionise the field of comparative religious studies in the same way that genetic databases have revolutionised the field of evolutionary biology.

## Introduction

Scholars have long attempted to explain religion from a naturalistic perspective [1]. Evolutionary theory offers a powerful framework with the potential to unite and inspire scholars from the social and biological sciences to generate and test hypotheses about the origin and functions of religion [2,3]. A wide range of evolutionary theories of religion have been proposed and debated [4–8]. These debates include whether religions are adaptations or by-products [5,9], which if any features of religions, such as “Big Gods”, “Costly Rituals”, and “Religious Elites”, provide benefits to individuals and groups [10–12], and whether functional features are recent or arose deep in the hominin lifeway [13]. However, progress in the evolutionary study of religion has been hindered by the absence of rigorous scientific tests. Proponents of one position or another have often based their arguments on hand-picked case studies selected from qualitative ethnographic records [3], or problematic quantitative datasets with limited variables on supernatural belief and practice [14]. Though qualitative data is a useful for generating hypotheses, testing hypotheses requires large datasets and rigorous quantitative methods.

At present, there are no other functioning quantitative cross-cultural databases that focus specifically on supernatural beliefs and practices. For this reason, many important evolutionary hypotheses about religion remain largely untested. Two general quantitative cross-cultural databases include religious variables—the Ethnographic Atlas (EA), and a database based on a subset of these cultures known as the Standard Cross Cultural Sample (SCCS) [15,16]. Though the religion variables in these databases have formed the basis for a number of studies on the evolution of religion [17–19], these databases have three major limitations.

First, the EA and SCCS contain a limited range of variables about supernatural beliefs and practices. The EA contains only two variables on supernatural beliefs: one concerns high gods, and the other concerns trance states. This limitation may account for the central focus that has been placed on moralising high gods in recent literature [17–20], despite such gods having arisen recently and being atypical of religion globally [14,21], and the focus of older literatures on a wide array of religious practices, institutions, and norms [22–24]. The SCCS includes a greater number of religious variables, but it suffers from high rates of missing data, and the variables focus primarily on the presence of specific religious practitioners, limiting the range of hypotheses that can be tested.

Second, the cultures sampled in the EA and SCCS are related through common ancestry and processes of cultural diffusion such as trade, missionisation, and colonisation [25,26]. Cultural diffusion can result in a wide range of cultural traits being transmitted. For example, colonisation often resulted in religious, political, and economic systems being imposed on cultures [14]. When standard correlational methods are applied to data from the EA and SCCS, associations between traits could be due to inheritance from a common ancestor, patterns of cultural diffusion, or a causal link between the traits–the difficulty of distinguishing between these explanations has come to be known as Galton’s Problem in the social sciences [27,28]. Recently, statistical methods have been developed that can address Galton’s Problem by controlling for common ancestry through phylogenetic models [29] and controlling for cultural diffusion through modelling social networks and geographic proximity [30–32]. However, the EA and SCCS samples were not built to accommodate such methods, and researchers face a number of difficulties when applying them to these databases [14,25,26]. For example, the EA and SCCS use global samples for which there are currently no credible cultural phylogenies, leading researchers to control for common ancestry based on crude language family assignments. However, controlling for language family does not take into account the relationship between language families, nor the internal relationships within these families, and so does not adequately account for the effects of common ancestry [25,33].

Third, though both the EA and SCCS specify the year to which a culture profile applies, neither database systematically distinguishes between the traditional state of a culture and its more recent state. For example, in the EA moralising high gods are coded as absent among Samoans and present among the Chamorro [15], despite the fact that this belief is not traditional to either culture, and both converted to Christianity before the specified time focus [34,35]. Supernatural beliefs are more richly and systematically profiled in the SCCS, however the time focus of these profiles has been decided primarily on the basis of the availability of detailed cultural and ethnographic records [16]. Consequently, some cultures have been coded on the basis of their traditional states, while others have been coded according to their more recent states.

## The *Pulotu* Database

Early European encounters with the striking diversity of cultures found throughout Austronesia inspired the modern academic study of religion as a comparative enterprise [36]. Despite the substantial documentation of cultural variation across Austronesia, however, there has yet to be any systematic database organizing data in a form amenable to rigorous quantitative analysis.

We have created a novel, purpose-built database to test evolutionary hypotheses of religious belief and practice using a sample of 116 Austronesian cultures. The database is named *Pulotu*, a reconstructed Proto-Polynesian term for ‘abode of the gods’ [37]. We sourced cultural data from a range of existing resources, such as ethnographic accounts, early explorers’ records, government statistics, cultural encyclopaedias and the Human Relations Area Files [38]. The database includes variables on the traditional states of cultures, the contemporary states of the cultures, and the events that occurred between these time foci. *Pulotu* is freely available at www.pulotu.com.

The Austronesian sample on which *Pulotu* is based has three key advantages over other major cultural families. First, Austronesian cultures and the physical environments that they inhabit are remarkably diverse. The cultures in our sample populate a vast geographic area, ranging west to Madagascar, east to Rapa Nui, north to Taiwan and south to New Zealand—a region spanning over half the world’s longitude and over a third of its latitude. The geographic settings of these cultures range from the tiny 1.1 km^2^ atoll of Kapingamarangi, to the fertile 10,458 km^2^ volcanic archipelago of Hawaii up to the 821,400 km^2^ continental island of New Guinea. The cultures themselves are no less diverse. In their traditional states, some cultures, such as the people of Rapa Nui, subsisted primarily on crops [39], whereas others such as the Savunese subsisted primarily on gathering [40], and others such as the Moken subsisted primarily on traded goods [41]. In terms of society, culture, and history, the Austronesian peoples were no less varied. Social organisation ranged from the small big-man-led villages of the Lak and Choiseulese [42] to the large, politically complex states of the Southern Toraja and Hawaii [43]. Some Austronesian societies such as the Tikopia were peaceful [44], whereas others such as the Waropen were in a constant state of war [45]. A wide spectrum of supernatural beliefs is presented across Austronesia, including localised nature spirits, the spirits of recent ancestors [46], as well as creator gods with ultimate power over the universe [47,48]. The importance placed on each type of God also varied greatly across Austronesian cultures. Rich social, cultural, and ecological variation is vital for reliably testing functional evolutionary hypotheses.

Second, family trees of Austronesian culture constructed using cognate data from the Austronesian Basic Vocabulary Database (ABVD) offer good proxies for the histories of these cultures [49], enabling the application of phylogenetic methods to the study of social phenomena [50]. Advantages of phylogenetic methods include the power to (a) address Galton’s Problem by modelling common ancestry [29], (b) infer the features of prehistoric cultures, such as proto-Austronesian [51], and (c) identify coevolution between traits, including the direction of causality, by inferring the temporal ordering of the traits [21].

Third, many cultures in Austronesia, particularly those in Oceania, had minimal contact with other cultures until the arrival of European explorers. The relative geographic isolation of such cultures has reduced the effects of cultural diffusion, and has led some to suggest that this region represent a natural laboratory for cross-cultural research [52]. Soon after European contact, the traditional states of these cultures were well documented, providing a rich resource base for the *Pulotu* database.

### The *Pulotu* Sample

The diversity of physical environments, social structures and supernatural belief systems presented in the *Pulotu* sample, combined with the ability to apply powerful phylogenetic methods, renders *Pulotu* an ideal database for testing functional hypotheses of religion. *Pulotu* contains 116 Austronesian cultures, and we have applied three principles when selecting these cultures. First, to facilitate the use of phylogenetic methods, priority has been given to cultures that speak one of the 400 languages found in Gray, Drummond, and Greenhill’s [50] phylogenetic tree. Second, we have ensured a representative sample by selecting cultures that are phylogenetically and geographically diverse. Our sample includes cultures from all major cultural groupings within Austronesia and all major geographic regions. Third, cultures have been excluded from our sample if there was major influence by a world religion prior to the collection of detailed ethnographic records. In Oceania, conversion to European monotheism has generally occurred within the last century and a half, and the state of cultures prior to conversion was often well-documented. However, there are parts of Indonesia that are known to have been in contact with Hindu, Muslim, and Buddhist cultures since at least the 10^th^ century [53]. In the cases of Bali and Java, major influence by Hinduism and Islam respectively occurred in historic time, and the earliest ethnographic records allow little to be inferred about the traditional religions of these cultures [54]. Excluding these cultures minimises the effects of diffusion and more accurately represents the diversity of traditional Austronesian supernatural beliefs and practices. Though cultures that were substantially influenced by a world religion, such as Bali and Java, have been excluded from our sample, cultures that were subject to minor influences have been included. Cultures have been categorized as having minor influence if there is evidence of diffusion from a world religion, yet the culture has maintained a distinct and unique belief system of their own. The Ngaju, who inhabit the remote and rugged regions of Central Kalimantan in Borneo, are an example of a culture with minor influence. In the traditional time period, Ngaju worship focused on two central deities; Kaloa, who owned the world’s surface and took the form of a one-breasted toad, and Hatara, who was god of the sky [55,56]. Though Kaloa appears to be of local origin, and Hatara as worshiped by the Ngaju was a unique god, it has been suggested that the name Hatara is a borrowing from the supreme Hindu god Batara Guru [55]. This example illustrates how influence by a world religion may have occurred, though without extensive effects. In cases similar to this example, external influence has been documented in the section of the database titled ‘Cultural Isolation’. Of the 116 cultures sampled in *Pulotu*, 93 (80%) show no signs of influence by a major world religion during the traditional time focus, indicating that the majority of cultures in our sample were not substantially influenced by world religions at the point early ethnographic records were taken.

The units of analysis in *Pulotu* are cultures. A culture has been defined as a group of people living in a similar physical, social and economic environment that speak mutually intelligible languages and have relatively homogenous supernatural beliefs and practices. An example of the kind of minor variation we permitted when categorizing cultural groupings are the various myths of man’s creation found in Kédang culture. Such myths follow a similar narrative in agreeing that man emerged from some other physical form; yet the myths also differ about whether this form was a goat, bamboo or a rock [57]. The cultural groupings described in ethnographies generally meet our definition of a culture. In some situations, generalisations about broader cultural groups, such as the cultures of the Vanuatu archipelago, are made in the ethnographic literature. While today such cultures form a political and cultural unit, before European contact there was major variation in their supernatural beliefs and practices, including the kinds of supernatural beings worshiped. For the people of Mota, a major focus of worship was the spirits of stones, trees and animals, which were of little or no importance to the people of Southern Malekula who instead focused on the worship of deceased ancestors [46,58]. In cases such as this where major variation between cultural groups exists, the groups are treated as separate cultural units.

The cultural groupings referred to within *Pulotu* can be identified by ethnonyms, their geographic coordinates, and the ISO-639-3 and ABVD language identification codes of the languages that they speak. In 24 of the 116 cultures sampled more than one dialect or language can be linked to a single culture. An extreme example is the people of Choiseul Island in the Solomon Islands, who can be linked to twelve distinct but closely related languages [59]. When more than one language or dialect listed in the ABVD was spoken by the culture, all have been listed in *Pulotu*.

### Sections and Variables of *Pulotu*

**Fig 1:**
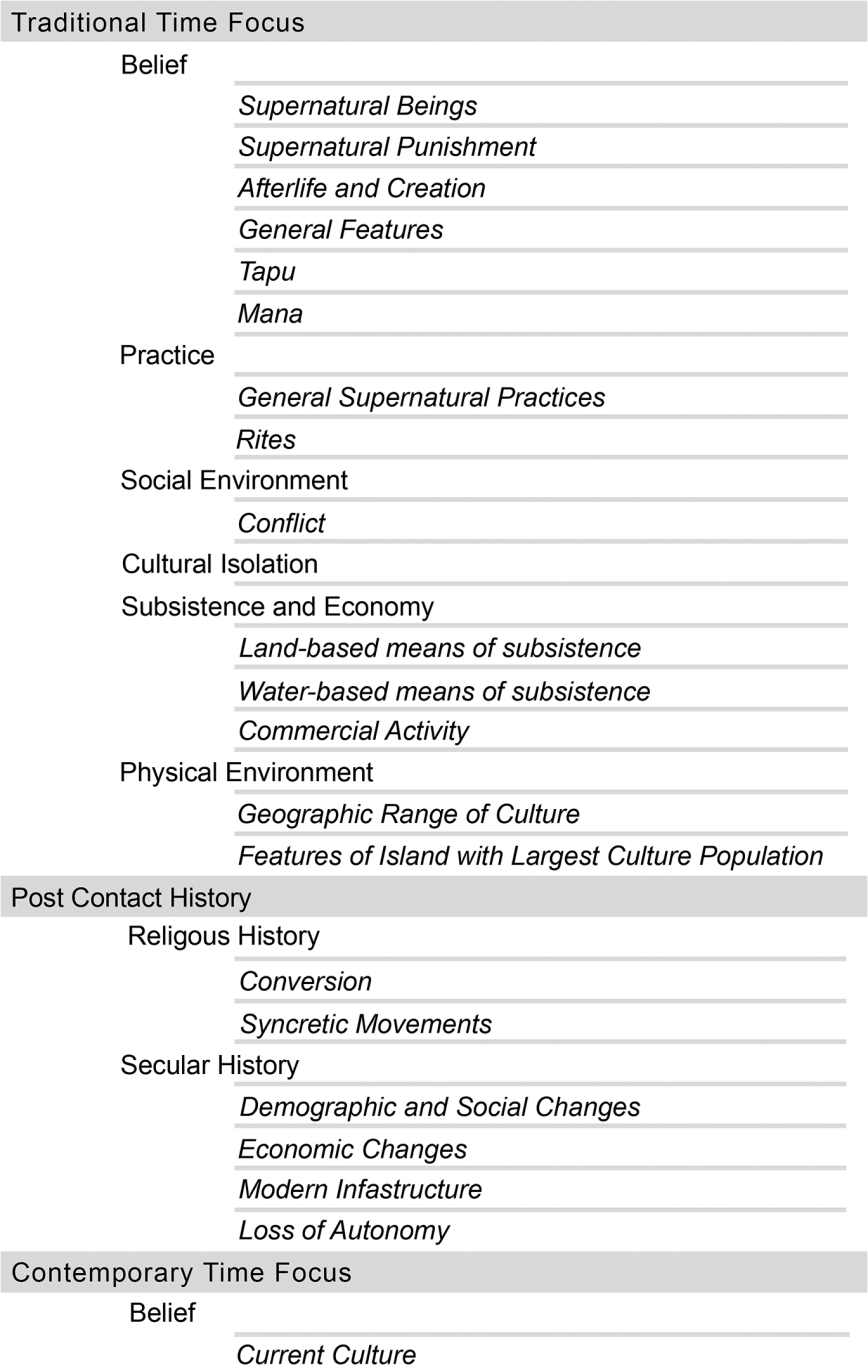
*Pulotu* Variable Structure. Organisation of variables in *Pulotu*, structured into time foci (level 1), sections (level 2) and sub-sections (level 3).

The variables in *Pulotu* are divided into three major domains: traditional state, post-contact history, and contemporary state (highlighted in Fig 1). The exact time period that these sections cover depends on the history of the culture itself. In the case of the Chamorro, major cultural change due to Spanish influence occurred in the 17^th^ century [60]. In cases such as the Central Tagbanwa, substantial religious influence did not occur until the 20^th^ century [61]. The precise period covered by the time focus is specified in *Pulotu* for each culture, and the variables in each domain apply strictly to that period. It is possible for a culture to be coded as lacking belief in a moralising high god in the traditional time focus yet also coded as predominantly Christian in the contemporary time focus. The traditional time focus covers a time period prior to large-scale contact and influence by cultures practicing world religions, such as Christianity, Islam or Hinduism. Coding for a period of time rather than a single point in history means that information about intermittent activities, such as contact and warfare with other cultures, can be more accurately coded. The contemporary state of the culture specifies the state of the culture in the year 2014, the year that the first database was finalised. The post-contact history domain details the period of time between the end of the traditional time focus and the beginning of the contemporary time focus. Each domain is divided into sections (shown in plain text in Fig 1), that are further divided into sub-sections (shown in italics in Fig 1).

### Coding Principles

To ensure uniform data entry within *Pulotu*, a number of coding principles have been applied to the selection of time foci, data uncertainty, and the selection of appropriate sources of cultural information.

#### Selection of time foci

The three major domains of *Pulotu*, the traditional state, contemporary state and post contact history, have been chosen on the basis of when major influence from cultures practicing world religions occurred, as well as by the availability of high-quality ethnographic sources. For the traditional state time focus, a range of 25 years, approximately one human generation, has been used. This range is desirable because it provides a long enough interval of time to include infrequent life events, but is short enough to minimise large scale cultural change. In the cases of Nuaulu, Ngaju, and Baduy, a period of time longer than 25 years has been specified because the required ethnographic records of the traditional culture were collected over a longer period of time. In every case, the range of dates for source materials is precisely described in *Pulotu*. The contemporary state concerns the state of a culture in 2014. Selection of a year is appropriate because the contemporary state domain contains no variables on the long-term frequencies of cultural events. Additionally, increasing trade, communication, travel, and technology means that cultures likely undergo greater rates of social and cultural change, which makes a shorter time interval more appropriate. The post-contact history is categorized as the time between the end of the traditional time focus and the beginning of the contemporary time focus. Precise intervals for each culture covering the post-contact period are also presented in *Pulotu*.

#### Sources

Many resources have been used to code cultures. We have given priority to primary ethnographic accounts published in peer-reviewed journals and books, as well as official government census records. When primary sources have been insufficient to code a variable in *Pulotu*, secondary sources, such as cultural encyclopaedias and historical summaries, have been consulted. The mean number of sources used to code a single culture in *Pulotu* is 20, while the minimum is 8, and the maximum is 41. Wherever possible, multiple independent sources have been used to code each variable.

#### Uncertainty in Ethnographic Data

Austronesian cultures were impressively well-documented by early explorers and ethnographers. However there are cases of uncertainty arising from contradictory claims in sources, ambiguity in the wording of ethnographies, and variation within cultures. Although cultural units, as we have operationalised the term, are by definition largely homogenous, minor variations exist in many cultural units. Where variation in a culture in respect to a categorical variable is observed, such as the contemporary dominant world religion, the most representative state of the cultures has been coded. For example, today the world religions of Islam and Christianity are both practiced among the Kédang, though as Christianity is practiced by a clear majority, the Kédang were coded as being Christian in the contemporary state [62]. When no one state could be reliably identified as most representative of the culture, the data have been marked as missing for that variable. If variation was observed in a culture in respect to an ordinal variable, such as the number of levels of jurisdictional hierarchy beyond the local community, the highest level found within the culture has been coded. For example, in Kiribati culture, some communities had no levels of political authority beyond the local community—these communities were made up of multiple families settled around a maneaba (meeting house), where a local council met to make decisions [63,64]. However, in the northern islands there was a level of political authority beyond the local community in the form of chiefs that exercised authority over multiple maneaba, and so Kiribati has been coded as having one level of political authority beyond the local community. Coding the highest level of political authority avoided the need to introduce missing data when no single state was uniformly representative across the culture.

#### Coding Procedure

Cultural profiles were completed by a trained coder, after gathering and reviewing available resources. A detailed codebook providing specific information about how to code each variable is included in S1 File and terms are clarified in the online glossary. Each coding decision includes references to the resources used to code it, which can be seen by clicking the source link next to a response in the culture profile. These references provide a link to additional resources for those interested in a particular variable, and allows others to replicate our coding decisions. Every cultural profile in *Pulotu* has been reviewed and replicated by at least one other coder. In cases of disagreement at least one additional coder was included. All coders reviewed all relevant literature and discussed each coding decision. If a clear mutual agreement was not reached, the data was marked as missing for that variable. Because *Pulotu* is freely available to both scholars and the general public, researchers with expertise on particular cultures will be able to review and verify all our coding decisions and suggest revisions through the contacts page.

### Website Features

*Pulotu* is open access and allows users to download the database, find background information on the project, and use a range of unique features for visualising and locating cultural information. By making *Pulotu* an open access database coding decisions can be cross-checked by experts, and previous research results can easily be verified and replicated.

#### Locating Cultures

**Fig 2:**
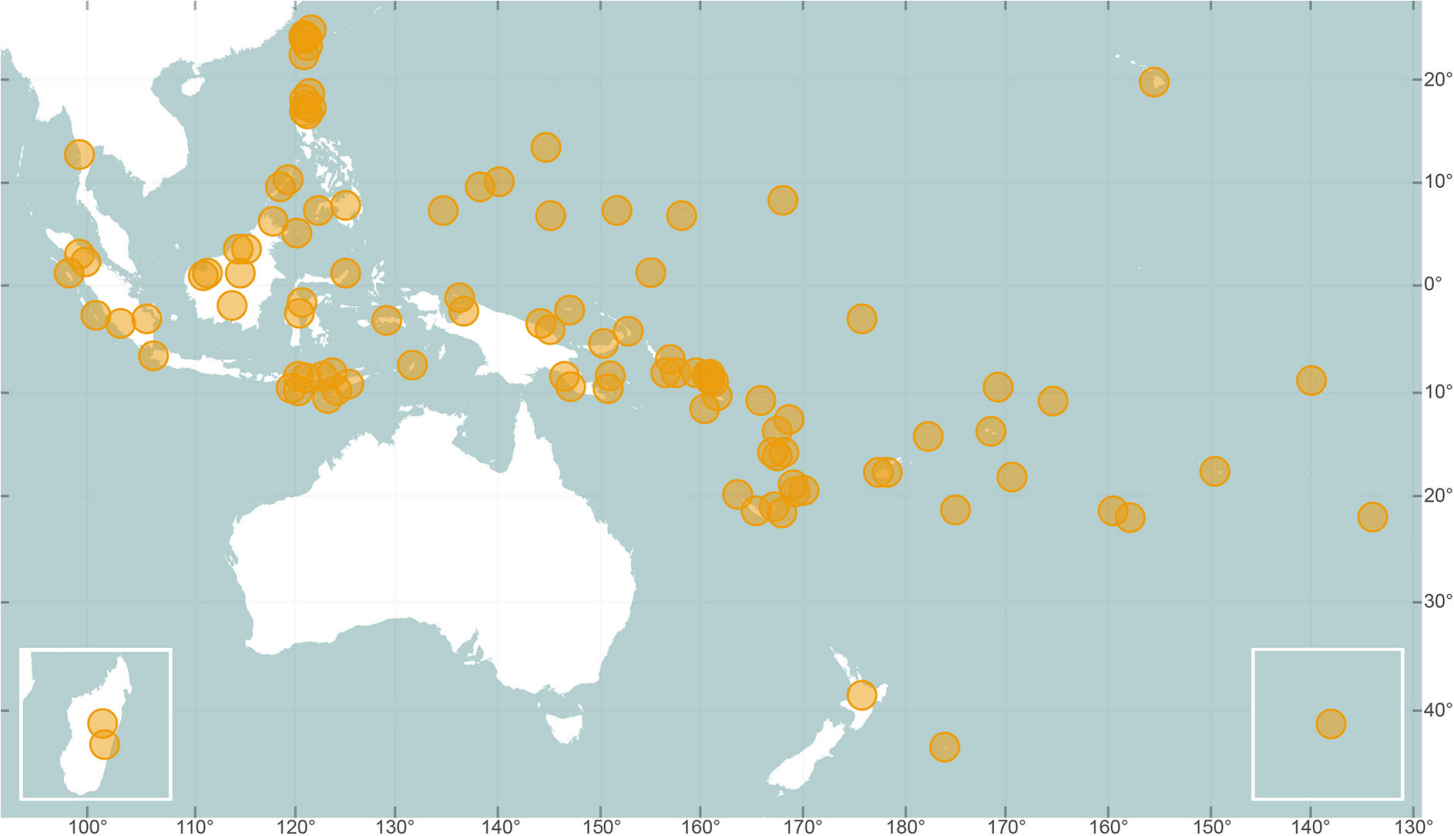
Geographic distribution of cultures in the *Pulotu* database. Madagascar is in the lower left box and Rapa Nui is in the lower right box. The axes represent the latitude and longitude of cultures in decimal degrees. Figure made using vector map data from Natural Earth.

Culture profiles can be reached through one of four paths; (1) the search bar at the top right of every page, (2) in the list of culture names, which includes alternative spellings and ethonyms under the ‘Cultures’ section, (3) by locating a culture on the map in the ‘Cultures’ section (Fig 2), or (4) by plotting variables on a map and then clicking the culture’s location in the ‘Compare Cultures’ section.

#### Culture Profiles

Each culture profile contains a list of the variables, responses to those variables, a bibliography, a map of the culture’s physical location, and links to external websites such as corresponding language entries in the Austronesian Basic Vocabulary Database and Ethnologue [65]. Variables are organised into domains, sections and sub-sections (see Fig 1 for the structure). Clicking on a section title expands the contents and displays a list of concise variable titles, responses, and a link to the sources used to code the variable. A detailed description of the variable title is provided in the glossary.

#### Comparing Cultures

**Fig 3:**
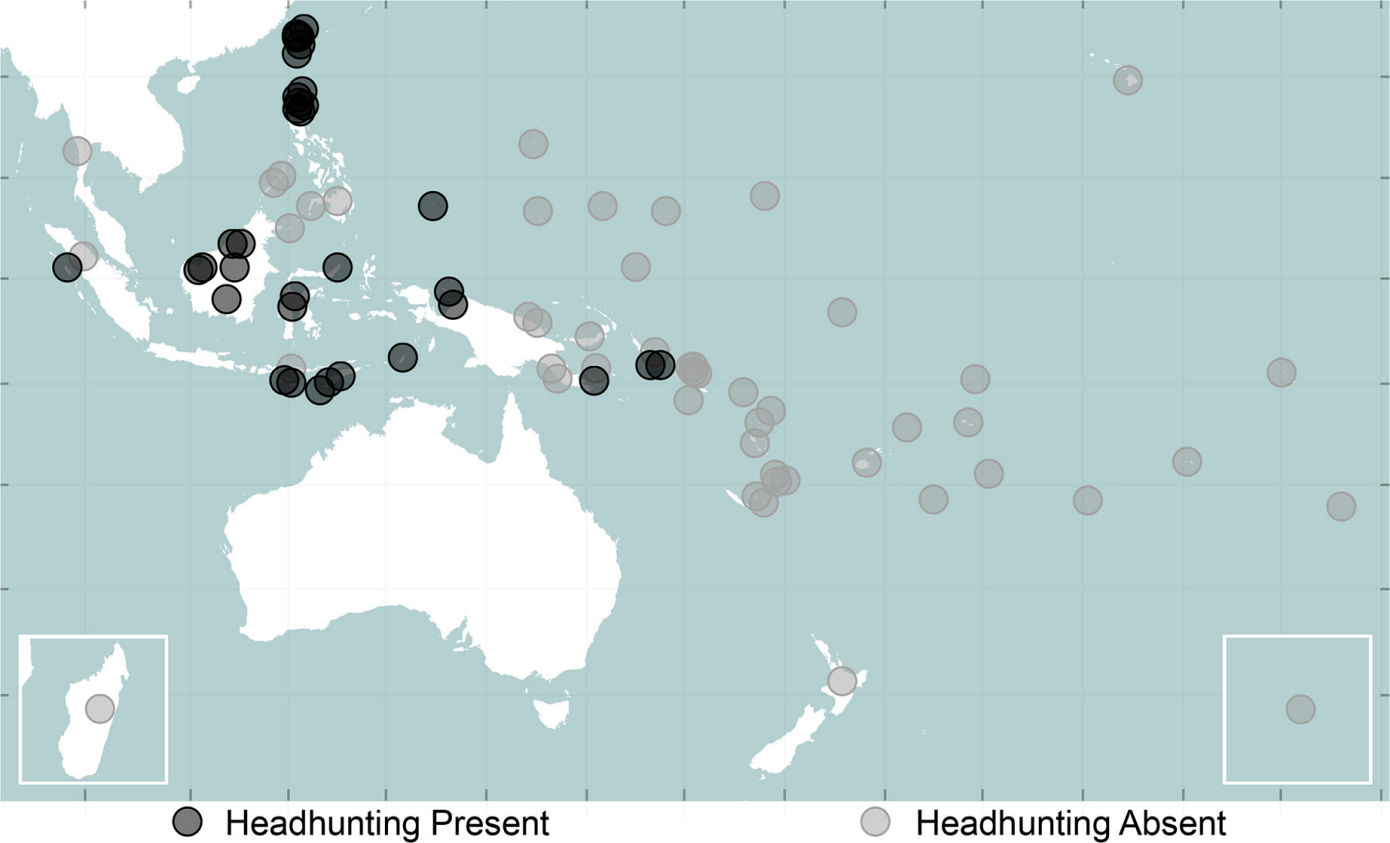
Geographic distribution of headhunting in Austronesia. Madagascar is in the lower left box and Rapa Nui is in the lower right box. The axes represent the latitude and longitude of cultures in decimal degrees. Figure made using vector map data from Natural Earth.

A notable feature of *Pulotu* is the ability to visualise the geographic distribution of traits (Fig 3 provides an example). The *Compare Culture* section contains a map and list of categorical variables. Selecting a variable displays circles in the locations of cultures with available data, and the colour of these circles represents the state of that culture for the given variable. A key provides the possible variable responses and their corresponding colours. Displaying variables on a map allows users to quickly and easily identify the geographic clustering of traits, which can be used to inform initial hypotheses about their spread and origin.

#### Downloading the Database and Codebook

The database can be downloaded as a tab-delimited txt file, which is compatible with a wide range of statistical software packages such as R, SPSS, Python, and Excel. Names of cultures are found at the beginning of each row, and for each variable there are two columns. The first column indicates the state of the culture for that variable, the second provides references to the sources used to inform that coding decision.

#### Future Development

*Pulotu* will remain publically available for the foreseeable future, and additional variables will continue to be added and released. We welcome user contributions, including suggestions for new variables, the addition of new cultures, and suggested corrections. The procedure for making these suggestions is outlined in the Frequently Asked Questions section of the website.

### Software and Web Hosting

The *Pulotu* website is implemented using the programming language *Python* and the open-source web-development framework *Django* (http://www.djangoproject.com). The user interface is implemented in HTML and javascript, and allows public users to search for and display the public data for each culture. The compare cultures feature was constructed with the OpenLayers mapping library (http://openlayers.org/). The user interface allows editors to maintain the database, add new cultural data, and download the database.

All cultural data in *Pulotu* is stored in Unicode format UTF-8 using the open-source relational database *PostgreSQL*. The core schema shown in S1 Fig illustrates how cultural data are normalised across the following eight core database tables:

1. The *Culture* table stores information about each culture, including the culture name, ethonyms, languages spoken by the culture, a brief description of the culture, and a foreign key link to the *Users* table identifying the person who coded the culture.
2. The *Language* table stores information about each language, including the language name, the ISO-639 identification code, and, where available, the language classification and ABVD code [65].
3. The *Source* table contains information about the sources used to code cultures. This includes the author list and year of source, the full APA reference, and any notes about the source. Again, each source has a foreign key link to the *Users* table identifying the person who added the source to the database.
4. The *Category* table contains information about the categories used to classify sections, including the category name and a flag for whether or not the category has an associated time focus.
5. The *Section* table contains information about the sections used to categorize questions, including the section name and any notes. Each section is linked via foreign key to a category in the *Category* table.
6. The *Question* table stores information about all the survey questions. Each question is linked to a section and subsection by foreign key in the *Section* table. Questions also include information on the response type (text, integer, float, or option), the question number, the question itself and a simplified version of the question for public display, and any further information for coders. Questions also have a flag denoting whether or not the question is displayed to the public.
7. The *Response* table contains all of the coded responses. There are four different response types in *Pulotu*: text, integer, float, and option responses. Each response is linked via foreign key to a culture in the *Culture* table, a question in the *Question* table, and sources in the *Source* table. Responses also include information on the coder's certainty about the response, any coder's notes, and a flag denoting whether the response is coded as 'Missing Data'.
8. The *User* table stores user information and access credentials for editors.

## Phylogenetic Analysis of *Pulotu* Data

As mentioned above, a major advantage of the *Pulotu* database is that cultures are linked to a dated Austronesian language tree, enabling the use of phylogenetic comparative methods. These methods can be used to control for Galton’s Problem and investigate a wide range of questions that traditional statistical methods cannot [29]. For example, phylogenetic methods can be used to infer the likely state of traits prehistoric cultures [66], test whether traits tend to evolve through a sequence of stages [51], and model the most likely pattern of co-evolution between traits [67,68]. Below we provide two examples of how phylogenetic methods can be applied to data from *Pulotu* to provide powerful insights into the evolutionary histories of cultures.

### Example 1: The Coevolution of Moralising High Gods and Political Complexity

In our first example we discuss the findings of a recent study that used Pagel’s *Discrete* software [67] to test whether concepts of moralising high gods co-evolved with political complexity in traditional Austronesian cultures [21]. Moralising high gods are powerful creator gods believed to actively monitor and punish immoral behaviour. Previous cross-cultural comparative studies, using traditional correlational methods with data from the SCCS and EA, show a relationship between moralising high gods and measures of social complexity, such as political stratification [17–19]. This has been taken as evidence for the hypothesis that the fear of supernatural punishment helps build large cooperative societies [69,70]. The first issue with these previous studies is that the cultures in the EA and SCCS are known to be non-independent [25], and so violate the assumptions of the methods used. The second issue is that most of moralising high gods found in the SCCS and EA belong to Christian and Islamic cultures, and it is not known if the effects of these two closely related religions are representative of supernatural punishment beliefs in general [14]. A third issue is that correlations do not get at the direction of causality, so it’s not clear whether moralising high gods lead to political complexity, or instead political complexity lead to moralising high gods [14]. By combining a phylogenetic method with data on traditional Austronesian cultures, Watts et al. [21] were able to address these limitations, and test for different patterns of coevolution between moralising high gods and political complexity.

**Fig 4:**
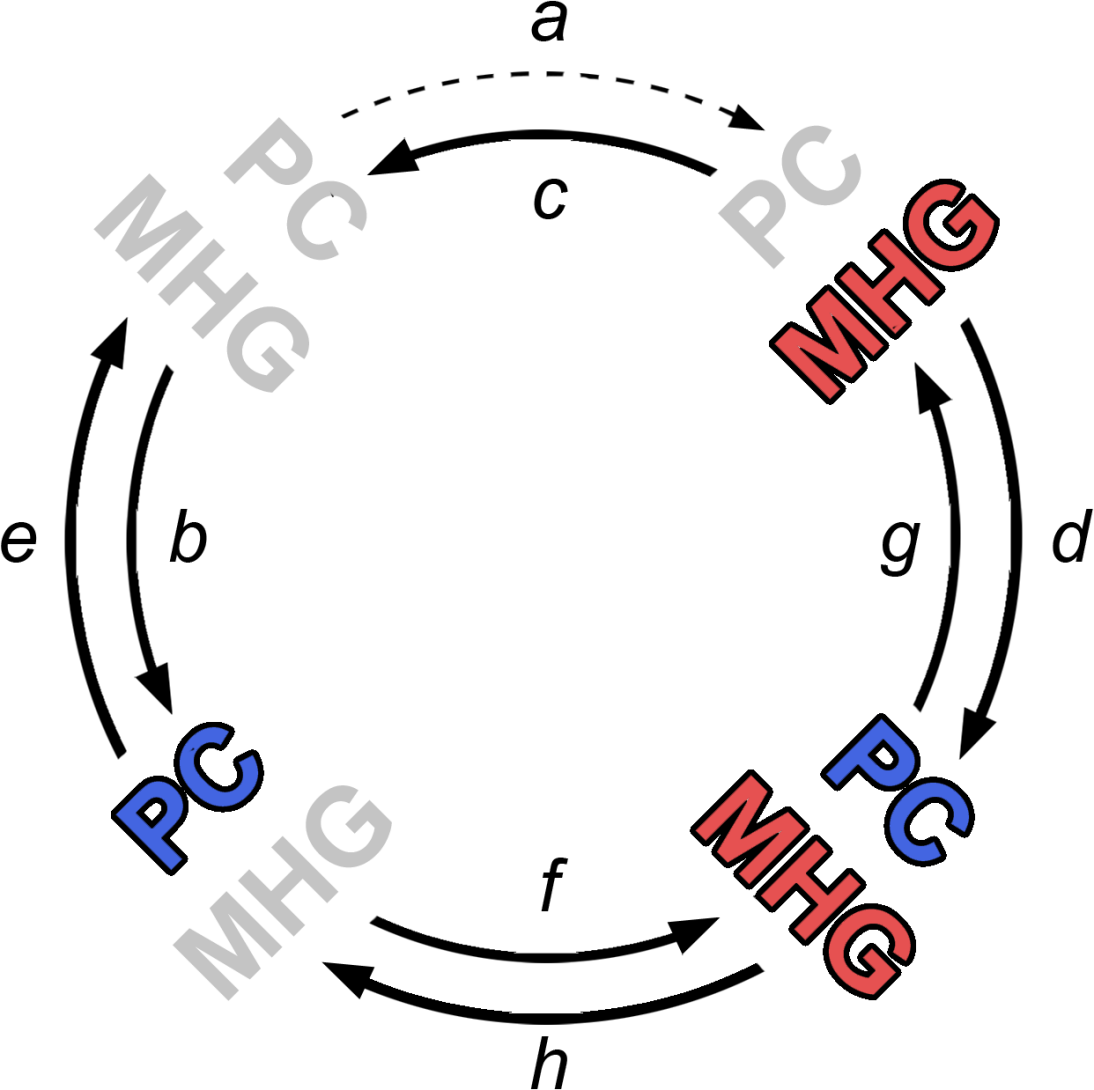
Co-evolutionary model of political complexity (PC) and Moralising High God (MHG) concepts in Austronesia. Grey represents the absence of a trait, and arrows represent rates of change.

The findings of this study indicate that moralising high gods co-evolved with political complexity, but the nature of this co-evolution challenges the causal model put forward in previous studies. If moralising high gods helped build politically complexity, then cultures with such concepts would be expected to gain political complexity at a higher rate than cultures without moralising high god concepts (in Fig 4 rate *d* should have been higher than rate *b*). However, no support for this prediction was found. Instead, politically complex cultures were inferred to gain moralising high god concepts at a greater rate than cultures with low political complexity (in Fig 4 rate *f* was greater than rate *a*). This indicates that belief in moralising high gods did not drive the evolution of political complexity, but instead may have spread to cultures after political complexity had already emerged.

Though moralising high god concepts are common in contemporary cultures, such concepts were scarce in traditional Austronesian cultures [21], and have recently spread recently through conversion to Christianity and Islam. These two closely related religions have a range of features, such as prescribed proselytization and a universal doctrine, which could explain their success [14]. More research is needed to test the effects of these religions in contemporary cultures. This example highlights the importance of systematically distinguishing between the contemporary and traditional states of cultures, and the power of using phylogenetic methods to infer the direction of causality.

### Example 2: Ancestral Reconstructing of Headhunting

**Fig 5:**
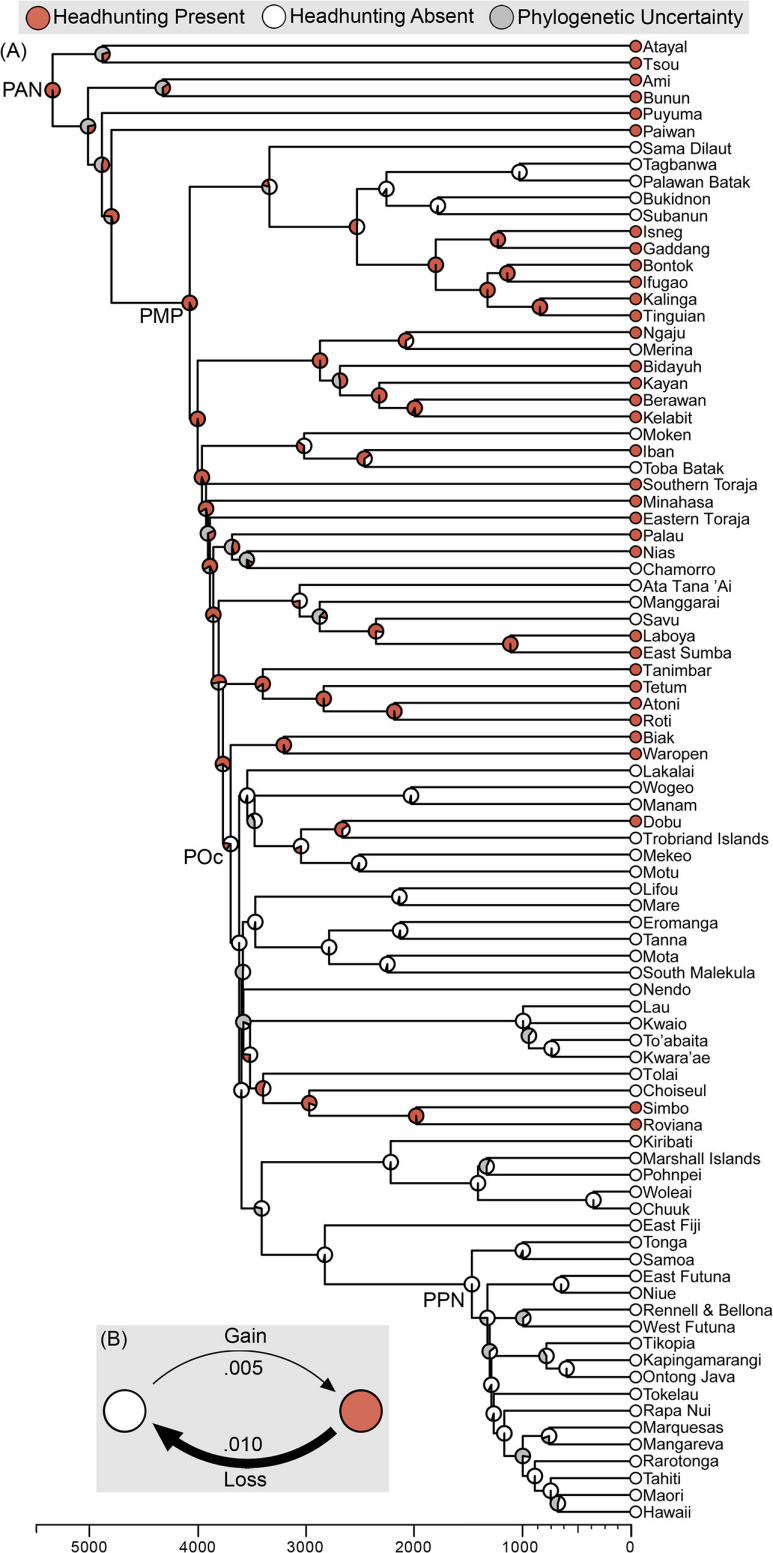
(A) Ancestral reconstruction of headhunting summarised on a consensus tree of Austronesian languages. Proto-Austronesia (PRA), Proto-Malayo-Polynesian (PMP), Proto-Oceanic (POc), and Proto-Polynesia (PPN) are labelled. The timescale along the horizontal axis indicates the number of years before the present day.(B) Transition rate matrix indicating the likelihood of a culture gaining or losing the practice of headhunting

In our second example we perform an ancestral state reconstruction to infer the likely prehistory of headhunting in traditional Austronesian cultures. Headhunting is the practice of killing people to obtain their heads. This practice was widespread in traditional Indonesian cultures (Figs 3 and 5A), and was typically believed to provide a supernaturally mediated reward, such as agricultural fertility, status in the afterlife, favour of spirits, protection of villages and general supernatural power. Though the practice of headhunting has been of great interest to anthropologists [71,72], little is known about the prehistory of this practice in Austronesia. Blust [73] conjectures that headhunting occurred at least 4,000 years ago in Proto-Malayo-Polynesian culture, and notes that while it may be even older, the linguistic reconstruction *kayaw alone provides insufficient evidence of a proto-Austronesian origin. Using the Multistate function in *Bayestraits*, and the language based trees of Gray et al. [50], we reconstructed the practice of headhunting in a sample of 88 cultures (See S2 File for Method details).

Our results provide strong support for Blust’s [73] claim that headhunting was practiced in Proto-Malayo-Polynesian culture (PMP, Fig 5A) and suggest an even earlier origin of headhunting in the common ancestor of all Austronesian cultures (PAN, Fig 5A). As well as being able to infer the state of proto-cultures, phylogenetic reconstruction is crucial to understanding the present distribution of traits. For example, the people of Nias are geographically isolated from other headhunting cultures, a fact that on its own might suggest that headhunting had recently arisen independently in the culture. However, taking into account the phylogenetic history of the culture indicates that the people of Nias are closely related to other headhunting cultures, and our ancestral state reconstruction indicates that the trait may have been inherited from a distant ancestor.

As well as supporting Blust’s hypothesis [73], this example illustrates the more general power of ancestral state reconstruction to identify patterns of cultural change across history. The rates in the transition matrix (Fig 5B) indicate that the chance of a culture losing headhunting is greater than the chance of a culture gaining headhunting. In the phylogenetic reconstruction (Fig 5A) numerous losses of headhunting can be seen throughout the history of Austronesia, including in Proto-Oceanic culture (POc, Fig 5A) and a substantial clade of the Philippines made up of the Tagbanwa, Palawan Batak, Bukidnon and Subanun. Our results imply that headhunting has also been gained at a number of points in the history of Austronesia, such as in the sister cultures of Simbo and Roviana located in the Western Province of the Solomon Islands. Though our model infers that Simbo and Roviana inherited the practice of headhunting from their common ancestor, this needs to be considered in the context of ethnographic literature. Simbo and Roviana engaged in regular trade with one another and performed headhunting raids on neighbouring islands such as Santa Isabel [74–77]. This suggests that another plausible history of headhunting in Simbo and Roviana is that headhunting arose independently and relatively recently in one of these cultures, and then diffused to the other. Phylogenetic models offer a powerful method of identifying large scale cross-cultural patterns which can then be contextualised using ethnographic, historical, spatial and archaeological data.

Future researchers might also use phylogenetic methods to test possible social functions of headhunting and identify why it was lost in Oceania. One hypothesis about the loss of headhunting is that the practice requires or is facilitated by iron weapons. Using linguistic evidence, Blust [78] proposes that Proto-Austronesian culture had knowledge of iron, and Proto-Malayo-Polynesian culture had knowledge of blacksmithing, suggesting that iron work is of similar antiquity to the practice of headhunting. However, iron technology is absent in Polynesian and other Oceanic cultures, indicating that iron tools may have been lost at a similar point of time to headhunting. *Pulotu* users could test the iron-dependency hypothesis using a phylogenetic co-evolutionary model. Additionally, regression-based phylogenetic methods could be used to test a range of other hypotheses about the social or ecological features associated with headhunting. Together these examples illustrate how using phylogenetic methods with *Pulotu* can provide powerful insights into the processes of religious and cultural evolution.

## Conclusion

*Pulotu* represents a new approach in cultural database design—it is the first quantitative database designed to tie cultures to phylogenies and include sections on the state of cultures at multiple time foci. The richly documented and remarkably diverse features of traditional Austronesian religions and cultures promise insights into the features of pre-modern cultures. Combining such data with phylogenetic methods enables scholars to identify general processes of religious and social evolution, and to resolve longstanding debates about the role of religion in human prehistory and the effects of religion on cultural development, stability and decline. For these reasons, applying phylogenetic methods to cross-cultural religious databases has the potential to revolutionise the field of evolutionary religious studies in the same way that phylogenetic methods and genetic databases have revolutionised the field of evolutionary biology.

## Supporting Information

S1 Fig Core Schema of the *Pulotu* database. (TIF)

S1 File *Pulotu* Codebook. This file contains the coding criteria and descriptions of the Pulotu variables.
(PDF)

S2 File Supporting Methods for Headhunting Example. This file contains additional information of the methods used in the headhunting example.
(DOCX)
