# Supplementary figures and images for: Pulotu: Database of Austronesian Supernatural Beliefs and Practices
Source: PLoS One. 2015 Sep 23;10(9):e0136783. doi: 10.1371/journal.pone.0136783 (PMC4580586; doi:10.1371/journal.pone.0136783)

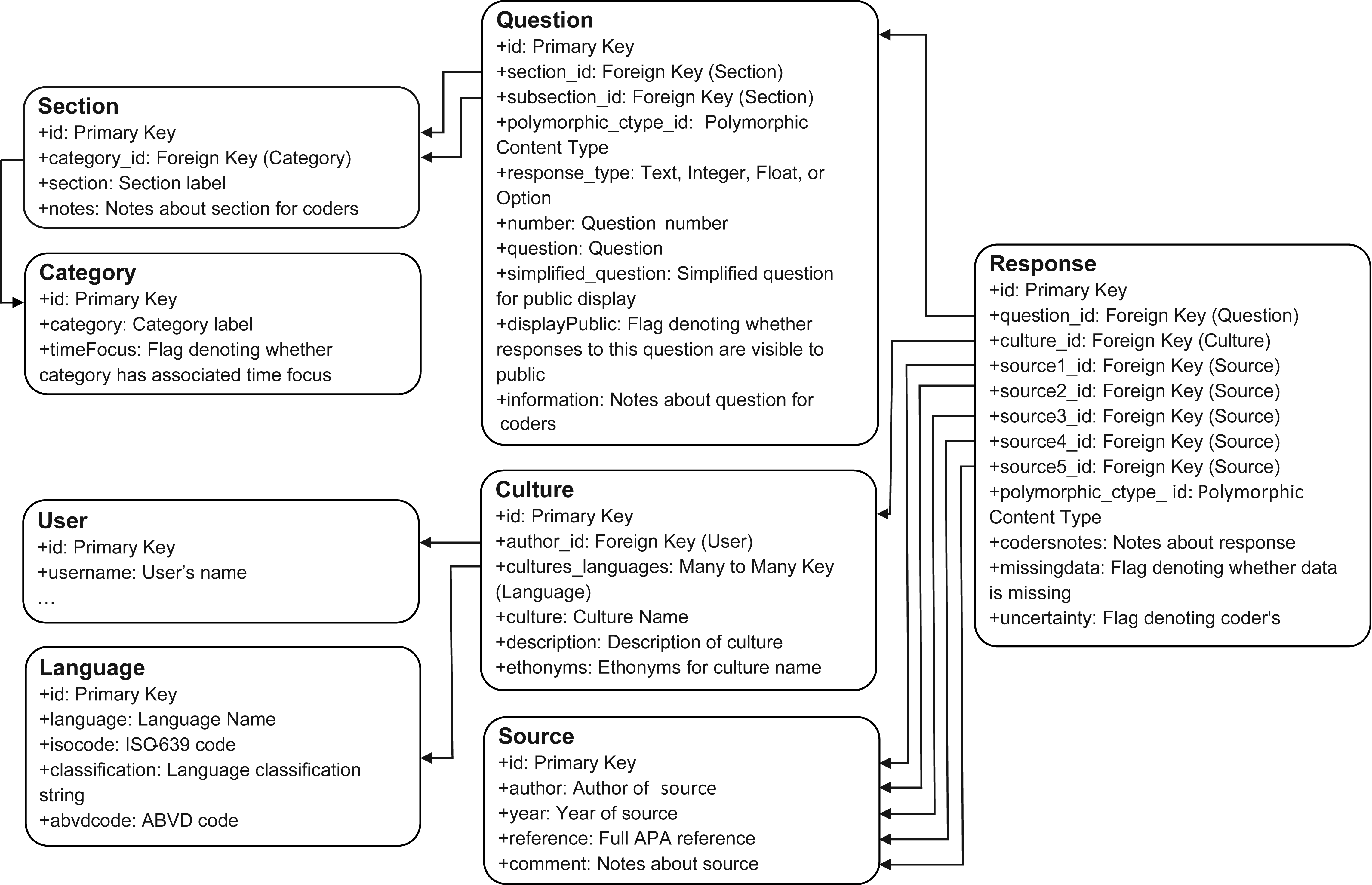

Supplement: S1 Fig — (TIF) [file pone.0136783.s001.tif]
