# Supplementary material for: Pulotu: Database of Austronesian Supernatural Beliefs and Practices
Source: PLoS One. 2015 Sep 23;10(9):e0136783. doi: 10.1371/journal.pone.0136783 (PMC4580586; doi:10.1371/journal.pone.0136783)
Supplement: S1 File — This file contains the coding criteria and descriptions of the Pulotu variables. (PDF) [file pone.0136783.s002.pdf]

Pulotu Codebook

Updated: 30/6/2015

Pulotu: Database of Austronesian Supernatural Belief and Practice

Authors: Joseph Watts, Oliver Sheehan, Simon J. Greenhill, Stephanie Gomes-Ng, Quentin D. Atkinson, Joseph Bulbulia and Russell D. Gray

Website: www.pulotu.com

Notes:

(1) There are gaps in the numbering of variables in this version of the codebook. The missing variables are either currently a work in progress, or are part of a research project and will be made public once this research is published.

(2) Variables derived from other sources as noted in brackets next to variable names. SCCS denotes the Standard Cross Cultural Sample.

(3) Additional clarification of terms is provided in the websites glossary.

Section 1: Indigenous Time Focus

v1. Indigenous State Time Focus

A time focus is the period of time to which information about a culture is applicable. Ideally, this section of the coding sheet should describe the culture as it was immediately prior to contact with cultures practising word religions (such as Christianity, Hinduism, and Islam). For islands with a long history of contact with the outside world, this may not be practicable – in cases such as these, the time focus should be the earliest period at which the culture can be reconstructed with a fair degree of certainty from ethnographic sources. The time focus should be stated in the form of a time interval of twenty-five years' duration, e.g. 1875-1900.

[Year range]

(?) Missing data

v2. Number of islands inhabited by culture

(1) One
(2) Two
(3) Three
(4) Four of more

(?) Missing data

v3. Distance to closest landmass inhabited by a different culture (km)

NOTE 1: A different culture, for the purposes of this coding sheet, is any culture other than the culture being coded. NOTE 2: The distance stated should be a minimum distance. For example, if the culture being coded occupied an archipelago, and the nearest other culture occupied an archipelago located to directly to the east, the distance given should be the distance from the easternmost island of the western archipelago to the westernmost island of the eastern archipelago. NOTE 3: If there was a different culture living on the same island, code this distance as "0".

[Distance in km]

(?) Missing data

v4. Distance to African or Asian mainland (km)

State minimum distance of whichever landmass was closest

[Distance in km]

(?) Missing data

v5. Latitude of culture's location on the island with the largest culture population (decimal degrees to one decimal place)

Positive for northern latitudes and negative for southern latitudes. If the culture occupied a large area, use the central point of this area. (Of island with largest population of culture or largest island if unknown)

[Latitude in decimal degrees]

(?) Missing data

v6. Longitude of culture's location on the island with the largest culture population (decimal degrees to one decimal place)

Positive for eastern longitudes and negative for western longitudes. If the culture occupied a large area, use the central point of this area. (Of island with largest culture population or largest island if unknown)

[Longitude in decimal degrees]

(?) Missing data

v7. Island type (island with largest culture population or largest island if unknown)

Only one of these options can be selected. If more than one of these processes was involved in the formation of the island, please code the most process that is most representative.

(1) Atoll (an oceanic low island of coral origin, e.g. Ulithi)
(2) Volcanic high island (an oceanic island of volcanic origin, e.g. Hawaii)
(3) Tectonic (an oceanic island formed by tectonic uplift, e.g. Macquarie Island)
(4) Continental island (an island situated on a continental shelf, e.g. Taiwan)

(?) Missing data

v8. Island Size (km^2)

Include any bodies of fresh water such as lakes, but do not include saltwater bodies such as lagoons. Consider atolls made up of smaller islets to be one island. (Of island with largest population of culture or largest island if unknown)

[Are in km^2]

(?) Missing data

v9. Maximum elevation (meters)

(Of island with largest population of culture or largest island if unknown)

[Elevation in meters]

(?) Missing data

v10. Estimate of culture population size

[Population]

(?) Missing data

v11. Estimated population of largest political community

A political community can be defined as a group of people sharing a common jurisdiction, or if no defined leadership is present as a group that makes important decisions by consensus on a regular basis. The largest such group situated wholly within the culture, or dominated politically by people from within the culture, should be coded.

(1) 99 or fewer
(2) 100-999
(3) 1,000-9,999
(4) 10,000-99,999
(5) 100,000 or more

(?) Missing data

v14. (No) conflict (social or political) within the local community (SCCS v 767)

(VARIABLE LABEL REVERSED) Conflict within the local community can include both interpersonal and intergroup conflict. Only conflict that poses a realistic threat to the cohesiveness of the community should be considered conflict for our purposes.

(1) Endemic (Conflict is frequent, is often violent, and is a pervasive aspect of daily life, e.g. feuding).
(2) High (Conflict is frequent and often violent, but is not a pervasive aspect of daily life.)
(3) Moderate (Conflict occurs frequently but is seldom violent, or is violent but occurs only occasionally.)
(4) Low (Conflict seldom occurs, and is almost never violent.)

(?) Missing data

v15. (No) internal warfare (between communities of the same society) (SCCS v 773)

(VARIABLE LABEL REVERSED) For the purposes of coding, internal warfare is warfare (i.e. lethal conflict between two or more groups of people) that takes place above the community level, but within the culture being coded. Thus warfare between two villages belonging to the same culture, or between two political communities (consisting of multiple villages) belonging to the same culture would be considered internal.

(1) Frequent, occurring at least yearly
(2) Common, at least every five years
(3) Occasional, at least every generation
(4) Rare or never

(?) Missing data

v16. (No) external warfare (with other societies) (SCCS v 774)

(VARIABLE LABEL REVERSED) For the purposes of coding, external warfare is warfare (i.e. lethal conflict between two or more groups of people) between members of the culture being coded and any group that is not considered part of the culture being coded. In larger cultures, the frequency of external warfare is likely to vary between subgroups. In cases such as this, the frequency that is typical for most political communities within the culture should be coded.

(1) Frequent, occurring at least yearly
(2) Common, at least every five years
(3) Occasional, at least every generation
(4) Rare or never

(?) Missing data

v17. (Low) contact with other societies (SCCS v 787)

(VARIABLE LABEL REVERSED) For the purposes of coding, contact with other societies is direct or indirect contact between one or more members of the culture being coded and one or more members of another culture. In larger cultures, the frequency of contact with other societies may vary between subgroups. In cases such as this, the frequency that is typical for most political communities within the culture should be coded.

(1) Frequent, through trade, warfare, travel, etc. (e.g. yearly or most years)
(2) Occasional but not often (e.g. once in a generation)
(3) Rare or never (less than once in a generation)

(?) Missing data

v19. Pre-Austronesian population

Based on Bellwood, Fox and Tryon (1995, p 109). Unless contrary evidence has come to light since 1995, all areas west of this line except for Madagascar should be coded as (2), and all areas east of the line should be coded as (0).

(0) Absent: No evidence of human occupation prior to Austronesian settlement
(1) Possible: Disputed / ambiguous evidence of human occupation prior to Austronesian settlement
(2) Present: Clear evidence of human occupation prior to Austronesian settlement

(?) Missing data

v20. Hindu / Buddhist influence on supernatural belief

Based on Cribb (2000).

(0) No evidence of influence and not in region of known contact (No evidence of Hindu / Buddhist influence on supernatural belief, and culture is unlikely to have had contact with Hindu or Buddhist societies at or prior to to the time focus in question.)
(1) No evidence of influence but in a region where contact is likely (No evidence of Hindu / Buddhist influence on supernatural belief, but culture is likely to have had contact with Hindu or Buddhist societies at or prior to the time focus in question.)
(2) Evidence of influence (Evidence of Hindu / Buddhist influence on supernatural belief.)

(?) Missing data

v21. Islamic influence on supernatural belief

Based on Cribb (2000).

(0) No evidence of influence and not in region of known contact (No evidence of Islamic influence on supernatural belief, and culture is unlikely to have had contact with Islamic societies at or prior to the time focus in question.)
(1) No evidence of influence but in a region where contact is likely (No evidence of Islamic influence on supernatural belief, but culture is known to have had (or is likely to have had) contact with Islamic societies at or prior to the time focus in question.)
(2) Evidence of influence (Evidence of Islamic influence on supernatural belief.)

(?) Missing data

v22. Christian influence on supernatural belief

(0) No evidence of influence and not in region of known contact (No evidence of Christian influence on supernatural belief, and culture is unlikely to have had contact with Christian societies at or prior to the time focus in question.)
(1) No evidence of influence but in a region where contact is likely (No evidence of Christian influence on supernatural belief, but culture is known to have had (or is likely to have had) contact with Christian societies at or prior to the time focus in question.)
(2) Evidence of influence (Evidence of Christian influence on supernatural belief.)

(?) Missing data

v24. Agriculture / Horticulture

Agriculture and horticulture refer to the cultivation of plants for food or other uses. In many parts of the Pacific, important food plants such as bananas and coconuts are cultivated but also gathered from the wild. Coders should try to identify which of these processes is operating, and to what extent, before coding.

(0) Absent (not practiced in the culture, or practiced but not a food source)
(1) Minor (forms a relatively insignificant part of diet)
(2) Medium (a significant food source, but not one of the most important sources of food)
(3) Major (one of the most important sources of food)
(4) Principal (more important than any other source of food)

(?) Missing Data

v25. Land-based gathering

Gathering is defined as the collection, as opposed to hunting, of wild foods. These foods can include parts of plants (e.g. fruits, nuts, roots), fungi, eggs, and small inverterbrates (e.g. insects, grubs, worms). Gathering does not include harvesting of fruit or nuts from trees that have been deliberately cultivated – this should instead be coded under “agriculture / horticulture”.

(0) Absent (not practiced in the culture, or practiced but not a food source)
(1) Minor (forms a relatively insignificant part of diet)
(2) Medium (a significant food source, but not one of the most important sources of food)
(3) Major (one of the most important sources of food)
(4) Principal (more important than any other source of food)

(?) Missing Data

v26. Animal husbandry as a source of food

Animals can be considered domestic if they are dependent, to an extent, on human care. Animals that were originally domestic but have become wild (e.g. feral pigs) should be coded under the hunting category.

(0) Absent (not practiced in the culture, or practiced but not a food source)
(1) Minor (forms a relatively insignificant part of diet)
(2) Medium (a significant food source, but not one of the most important sources of food)
(3) Major (one of the most important sources of food)
(4) Principal (more important than any other source of food)

(?) Missing Data

v27. Land-based hunting performed by individuals

For the purposes of this question and the next, hunting can be considered the killing of vertebrates for food. Invertebrates such as crabs and insects should be coded under the gathering category. Ethnographic literature is not always clear about whether individual or group hunting strategies are used. In cases of uncertainty, an educated guess should be made if possible. For example, small animals such as birds can be hunted by individuals, but large animals such as pigs usually require group hunting strategies.

(0) Absent (not practiced in the culture, or practiced but not a food source)
(1) Minor (forms a relatively insignificant part of diet)
(2) Medium (a significant food source, but not one of the most important sources of food)
(3) Major (one of the most important sources of food)
(4) Principal (more important than any other source of food)

(?) Missing Data

v28. Land-based hunting performed by one or more groups Absent (not practiced in the culture, or practiced but not a food source)

(0) Minor (forms a relatively insignificant part of diet)
(1) Medium (a significant food source, but not one of the most important sources of food)
(2) Major (one of the most important sources of food)
(3) Principal (more important than any other source of food)

(?) Missing Data

v29. Water-based gathering

For the purposes of this question, water-based gathering can be considered the collection of marine or freshwater plant foods (e.g. seaweed) and of small invertebrates such as shellfish and crustaceans.

(0) Absent (not practiced in the culture, or practiced but not a food source)
(1) Minor (forms a relatively insignificant part of diet)
(2) Medium (a significant food source, but not one of the most important sources of food)
(3) Major (one of the most important sources of food)
(4) Principal (more important than any other source of food)

(?) Missing Data

v30. Fishing and water-based hunting performed by individuals

Fishing includes the harvesting of true fish as well as large, motile invertebrates such as octopus and squid. Water-based hunting involves marine or aquatic mammals (e.g. seals, dolphins), birds (e.g. ducks), amphibians (e.g. frogs) or reptiles (e.g turtles). Opportunistic feeding on beached whales etc. does not belong in this category, but rather under gathering. As with hunting, it is not always clear in the ethnographic literature whether individual or group fishing strategies are being used. In these cases, an educated guess should be made if possible. For example, nearshore fishing can be carried out by individuals, but deepwater fishing usually requires a group approach.

(0) Absent (not practiced in the culture, or practiced but not a food source)
(1) Minor (forms a relatively insignificant part of diet)
(2) Medium (a significant food source, but not one of the most important sources of food)

(3) Major (one of the most important sources of food)
(4) Principal (more important than any other source of food)

(?) Missing Data

v31. Fishing and water-based hunting performed by one or more groups

(0) Absent (not practiced in the culture, or practiced but not a food source)
(1) Minor (forms a relatively insignificant part of diet)
(2) Medium (a significant food source, but not one of the most important sources of food)
(3) Major (one of the most important sources of food)
(4) Principal (more important than any other source of food)

(?) Missing Data

v32. Trade / wage labour as a source of food

(0) Absent (not practiced in the culture, or practiced but not a food source)
(1) Minor (forms a relatively insignificant part of diet)
(2) Medium (a significant food source, but not one of the most important sources of food)
(3) Major (one of the most important sources of food)
(4) Principal (more important than any other source of food)

(?) Missing Data

v35. Belief that forces of nature (eg. winds, tides, seasons, day and night) are controlled by or imbued with the supernatural

(0) Absent
(1) Present

(?) Missing data

v37. Belief in nature spirits

A nature spirit is a supernatural agent of non-human origin, who is not considered to be an ancestor, who has a narrow sphere of influence and concern, and who (a) is embodied in a particular feature of the natural world or natural phenomenon and is seldom or never encountered in any other form, and / or (b) resides in a particular natural landscape or type of natural landscape, and is seldom encountered anywhere else.

(0) Absent (do not feature in the belief system of the culture)
(1) Present, but not a major focus of supernatural practice
(2) Present, and a major focus of supernatural practice
(3) Present, and the principal focus of supernatural practice

(?) Missing data

v38. Belief in nature god(s)

A nature god is a supernatural agent of non-human origin, who is not considered to be an ancestor, who has a broad sphere of influence and concern, and who (a) is embodied in a particular feature of the natural world or natural phenomenon and is seldom or never encountered in any other form, and / or (b) resides in a particular natural landscape or type of natural landscape, and is seldom encountered anywhere else.

(0) Absent (do not feature in the belief system of the culture)
(1) Present, but not a major focus of supernatural practice
(2) Present, and a major focus of supernatural practice
(3) Present, and the principal focus of supernatural practice

(?) Missing data

v39. Belief in ancestral spirits

An ancestral spirit is the spirit of a deceased ancestor who was once a human or other corporeal being and continues to influence the lives of his or her living descendants. Unlike deified ancestors, ancestral spirits have a narrow sphere of influence and concern (usually confined to a single family), and are usually, though not always, the spirits of those who lived in the recent past.

(0) Absent (do not feature in the belief system of the culture)
(1) Present, but not a major focus of supernatural practice
(2) Present, and a major focus of supernatural practice
(3) Present, and the principal focus of supernatural practice

(?) Missing data

v40. Belief in deified ancestor(s)

A deified ancestor is the spirit of a human or other corporeal being, ancestral to at least a subset of people within the culture, who acquired godlike powers after death. Unlike ancestral spirits, deified ancestors have broad spheres of influence and concern (extending beyond single families to larger units such as tribes and societies), and are usually, though not always, believed to have lived in the distant past.

(0) Absent (do not feature in the belief system of the culture)
(1) Present, but not a major focus of supernatural practice
(2) Present, and a major focus of supernatural practice
(3) Present, and the principal focus of supernatural practice

(?) Missing data

v41. Belief in culture hero(es)

A culture hero can be defined as (a) a human, or some other corporeal being, who had godlike powers while living and whose actions explain the presence of a particular feature of the physical or social world, and / or (b) the spirit of any such being. Culture heroes are usually, but not always, believed to have lived in the distant past. Some culture heroes are also deified ancestors.

(0) Absent (do not feature in the belief system of the culture)
(1) Present, but not a major focus of supernatural practice
(2) Present, and a major focus of supernatural practice
(3) Present, and the principal focus of supernatural practice

(?) Missing data

v42.Belief in God(s)

A god is a supernatural agent with a broad sphere of influence and concern, who is not restricted to any one physical form or geographical area, and is not believed to have originated as a human or other corporeal being.

(0) Absent (do not feature in the belief system of the culture)
(1) Present, but not a major focus of supernatural practice
(2) Present, and a major focus of supernatural practice
(3) Present, and the principal focus of supernatural practice

(?) Missing data

v44. Belief in supernatural punishment for impiety

Impiety is behaviour that is directly offensive to gods or other supernatural agents – examples might include blasphemy, desecration of a sacred site, or failing to carry out rituals correctly.

(0) Absent
(1) Present

(?) Missing data

v46. Belief that one's actions while living can affect the nature of one's afterlife

(0) Absent (Actions while living do not affect the nature of one's afterlife)
(1) One factor in determining one's afterlife (Actions while living are one factor in determining the nature of one's afterlife)
(2) Principal determinant of one's afterlife (Actions while living are the principal determinant of the nature of one's afterlife)

(?) Missing data

v47. Belief that the actions of others after one has died (e.g. funeral rites) can affect the nature of one's afterlife Absent (Actions of others after one has died do not affect nature of one's afterlife)

(0) One factor in determining one's afterlife (Actions of others after one has died are one factor in determining the nature of one's afterlife)
(1) Principal determinant of one's afterlife (Actions of others after one has died are the principal determinant of the nature of one's afterlife)

(?) Missing data

v48. Myth of man's creation

(0) Absent
(1) Present, and evolutionary (man came from some other form of life)
(2) Present, and creationist (man was created by some agent in the same form as he is today)

(?) Missing data

v49. Myth of a primordial pair

A primordial pair is a male-female pair of supernatural beings who were present at the beginning of the world and are believed to be the parents of the gods and/or of the human race.

(0) Absent
(1) Present, and genealogically linked to humans now living

(2) Present, and genealogically distinct from humans

(?) Missing data

v51. Social hierarchy tapu

A tapu that mandates or forbids certain behaviours between people of differing social status.

(0) Absent
(1) Present

(?) Missing data

v52. Kinship tapu

A tapu that mandates or forbids certain behaviours between biological or classificatory relatives

(0) Absent
(1) Present

(?) Missing data

v53. Resource management tapu

A tapu that protects, either permanently or temporarily, an important communally-owned resource

(0) Absent
(1) Present

(?) Missing data

v56. Mana related to social influence or technical skill

e.g. mana means “prestige” Please provide a brief description in the Coder's Notes section.

(0) Absent
(1) Present

(?) Missing data

v57. Mana as a spiritual or religious concept

e.g. mana means “favoured by the gods”. Please provide a brief description in the Coder's Notes section.

(0) Absent
(1) Present

(?) Missing data

v58. Mana as a personal quality

In the culture in question, does it make sense to describe a person or other agent as being mana / having mana?

(0) Absent (skip questions 59 & 60)
(1) Present

(?) Missing data

v59. Mana and social status

(1) Unrelated (Mana is unrelated to social status.)
(2) Moderately associated (Mana is associated with social status, but high social status is not necessary in order to be mana or have mana.)
(3) Tightly coupled (Mana is closely linked to social status – only people with high social status can be mana or have mana.)

(?) Missing data

v60. Mana linked to genealogy

Does descent have a bearing on whether someone is or has mana?

(0) Absent
(1) Present, linked to paternal lines only
(2) Present, linked to maternal lines only
(3) Present, linked to both paternal and maternal lines

(?) Missing data

v61. Political and religious differentiation (SCCS v 757)

(1) Considerable overlap between religious and political leaders (Most religious leaders, or the most prominent religious leaders, are also political leaders)
(2) Some overlap (Some religious leaders, but not the most prominent religious leaders, are also political leaders, or religious and political leaders are distinct but political leaders exert considerable influence on religious leaders)
(3) No overlap (Religious leaders are distinct from, and independent from, political leaders)

(?) Missing data

v63. Headhunting

Headhunting is the practice of killing people for the sole or primary purpose of obtaining their heads.

(0) Absent
(1) Present

(?) Missing data

v64. Costly sacrifices and offerings

Costly sacrifices and offerings are defined as the presentation of an object of more-than-trivial value to a supernatural agent, which involves the forfeiting of that object, or a valuable part of that object, for any further use. Costly sacrifices and offerings may involve inanimate objects or living beings, but for our purposes human sacrifices should be excluded from this category. Funeral practices which invoke the destruction of the property of the deceased or the burial of valuable objects with the deceased should be included in this category, but only if there is an explicit or clearly implied belief that the objects will benefit the deceased in some way in the afterlife.

(0) Absent
(1) Present

(?) Missing data

v65. Size of largest ritual social group

A ritual social group is a group of people that (a) are under the same religious authority, or (b) conduct religious rituals together on a regular basis.

(1) Household or smaller
(2) Larger than a household, no larger than the local community (e.g. village)
(3) Larger than a local community, no larger than the largest political community in the culture
(4) Larger than the largest political community in the culture

(?) Missing data

v66. Tattooing

(0) Absent from culture
(1) Present in culture, but not as a rite or feature of a rite
(2) Present in the culture as a rite or feature of a rite

(?) Missing data

v67. Scarification

Scarification must involve deliberately inflicted permanent scarring, not superficial cutting.

(0) Absent from culture
(1) Present in culture, but not as a rite or feature of a rite
(2) Present in the culture as a rite or feature of a rite

(?) Missing data

v68. Piercing

(0) Absent from culture
(1) Present in culture, but not as a rite or feature of a rite
(2) Present in the culture as a rite or feature of a rite

(?) Missing data

v69. Genital Cutting

(0) Absent from culture
(1) Present in culture, but not as a rite or feature of a rite
(2) Present in the culture as a rite or feature of a rite

(?) Missing data

v70. Tooth Pulling

(0) Absent from culture
(1) Present in culture, but not as a rite or feature of a rite
(2) Present in the culture as a rite or feature of a rite

(?) Missing data

v105. Importance of Patrilateral descent

(1) Low (Patrilateral descent plays little or no role in determining social status and / or group membership.)
(2) Medium (Patrilateral descent plays an important role in determining social status and / or group membership, but is not the most important form of descent.)
(3) High (Patrilateral descent plays an important role in determining social status and / or group membership, and is more important than any other form of descent.)

(?) Missing Data

v106. Importance of Matrilateral descent

(1) Low (Matrilateral descent plays little or no role in determining social status and / or group membership.)
(2) Medium (Matrilateral descent plays an important role in determining social status and / or group membership, but is not the most important form of descent.)
(3) High (Matrilateral descent plays an important role in determining social status and / or group membership, and is more important than any other form of descent.)

(?) Missing Data

Section 2: Post-contact history

v71. Loss of autonomy during postcontact period

(1) Low (The cultural remained politically autonomous throughout the post-contact period.) (Skip to Q.71)
(2) Medium (The culture maintained some political autonomy throughout the post-contact period, but on at least one occasion during the post-contact period the culture entered into a political relationship with a foreign power that involved some loss of autonomy.)
(3) High (On at least one occasion during the post-contact period, the culture came under the political dominance of a foreign power, and this event resulted in considerable loss of autonomy.)

(?) Missing data

v72. Nature of loss of autonomy – voluntary vs. forced

Was autonomy ceded voluntarily or by force? If autonomy was lost more than once and differing levels of force were involved, please code the highest level of force used.

(1) Largely voluntary (Autonomy was ceded either voluntarily or as a result of pressure from other members of the same culture, without the threat of external force being necessary.)
(2) Partly voluntary (e.g. Autonomy was ceded voluntarily but under duress; some communities ceded their autonomy voluntarily but others were forced to do so by an foreign power.)
(3) Largely involuntary (autonomy was lost largely as a result of external force)

(?) Missing data

v73. Replacement-level immigration

Replacement-level immigration, for the purposes of coding, is migration of people from non-indigenous cultures (i.e. cultures that were not originally present in the area) into the ancestral territory of the culture being coded, at such a level that the cultural demographics of the area were changed substantially. "Immigrant population", in this case, refers both to immigrants from another culture and to their descendants, but excludes those who became absorbed into the indigenous population through intermarriage or acculturation.

(0) Absent (Replacement-level immigration did not occur – an immigrant culture was never present in the area, or was present but had a population size less than 10% of that of the indigenous culture.)
(1) Low (Replacement-level immigration occurred. The immigrant culture had a population size more than 10% of that of the indigenous culture at one time or another, but this figure never exceeded 50%.)
(2) Medium (Replacement-level immigration occurred. The immigrant culture had a population size more than 50% of that of the indigenous population at one time or another, but this figure never exceeded 100%.)
(3) High (Replacement-level immigration occurred. At one time or another during the post-contact period the immigrant culture had a population size more than 100% of that of the indigenous population.)

(?) Missing data

v74. Language shift

(1) Low (The indigenous language remained the sole or primary means of communication for most of the indigenous population for the duration of the post-contact period.)
(2) Medium (The indigenous language remained an important means of communication for the duration of the post-contact period, but at one time or another the indigenous population used a non-indigenous language to roughly the same extent.)
(3) High (At one time or another during the post-contact period, a foreign language became the sole or primary means of communication for most of the indigenous population.)

(?) Missing data

v75. Foreign education systems

i.e. education systems not indigenous to the culture

(1) Low (At no time during the post-contact period did more than 25% of indigenous children receive a basic non-indigenous education.)
(2) Medium (At one time or another during the post-contact period over 25% of indigenous children received at least a basic non-indigenous education, but this figure never exceeded 75%.)
(3) High (At one time or another during the post-contact period over 75% of children received some at least a basic non-indigenous education.)

(?) Missing data

v76. Foreign government systems

A foreign government system need not imply political dominance by foreigners. A country may be politically independent and be governed by indigenous people, but have a system of government that is of foreign origin.

(1) Absent (At no time during the post-contact period was the culture subject to a foreign government system.)
(2) Present, but of low importance (At one time or another during the post-contact period, the culture was subject to a foreign government system, but this government system never controlled most important decisions.)
(3) Present, and of high importance (At one time or another during the post-contact period the culture was subject to a foreign government system that controlled most important decisions.)

(?) Missing data

v77. Changes in means of subsistence

Means of subsistence can incorporate new features (e.g. new technologies) without being fundamentally changed.

(2) Low (Principal and major means of subsistence have not changed from their indigenous states.)
(3) Medium (Principal and major means of subsistence have changed from their indigenous states (e.g. through gain or loss of major and / or principal means of subsistence), but at least one of the major or principal means of subsistance has persisted.)
(4) High (No indigenous means of subsistence has remained major or principal.)

(?) Missing Data

v79. Exportation of goods to other cultures

(2) Absent (Exports were never part of the local economy.)
(3) Present but minor (At one time or another exports were part of the local economy, but were never a primary focus of economic activity for more than 10% of the population .)
(4) Present and substantial (At one time or another during the post-contact period, exports were a primary focus of economic activity for over 10% of the population.)

(?) Missing data

v80. Vehicles and roads

For the purposes of coding, motor vehicles are not considered to include boats.

(0) Absent (At no time during the post-contact period did most members of the culture have access either to roads or motor vehicles.)
(1) Present but rarely used (At no time during the post-contact period were motor vehicles used by a substantial portion of the population, but at one time or another most members of population had access to roads suitable for motor vehicles.)
(2) Present and widely used (At one time or another during the post-contact period, motor vehicles were used by a substantial proportion of the population.)

(?) Missing data

v81. Sea port

i.e. a wharf that can dock large foreign ships

(0) Absent (At no time during the post-contact period did most members of the culture have access to a sea port.)
(1) Present (At one time or another during the post-contact period most members of the culture had access to a sea port.)

(?) Missing data

v82. Air travel

(0) Absent (At no time during the post-contact period did most members of the culture have access to an airport or airstrip.)
(1) Present, local only (At no time during the post-contact period did most members of the culture have access to international air travel, but at one time or another most members of the culture had access to domestic air travel.)
(2) Present and long-distance (At one time or another, most members of the culture had access to international air travel.)

(?) Missing data

v83. Adoption of a world religion

Did a substantial proportion of the indigenous population adopt a world religion?

(0) Absent or minimal (At no time during the post-contact period did more than 5% of the indigenous population practice a world religion.) (Skip to Q.87)
(1) Present but minor (At one time or another during the post-contact period more than 5% of the indigenous population practiced a world religion, but this figure never exceeded 25%.)
(2) Present and major (At one time or another during the post-contact period more than 25% of the indigenous population practiced a world religion, but this figure never exceeded 75%.)
(3) Present and predominant (At one time or another during the post-contact period, at least 75% of the indigenous population practiced a world religion.)

(?) Missing data

v84. Resident missionary involvement in conversion process

Did resident foreign missionaries play a significant role in the conversion process?

(0) Absent (Foreign missionaries played no role, or only a minimal role, in the conversion process.)
(1) Present, and from Austronesian societies only (Foreign missionaries played an important role in the conversion process, but these missionaries were from other Austronesian societies only.)
(2) Present, and from non-Austronesian societies (Foreign missionaries, at least one of whom was from a non-Austronesian society, played an important role in the conversion process.)

(?) Missing data

v85. Use of force in conversion

Was a religion imposed on the culture by a colonial government / external cultural body? If more than one conversion took place involving varying levels of force, please code the highest level of force used.

(1) Low (most communities adopted the new religion either voluntarily or due to pressure from other communities of the same culture)
(2) Medium (e.g force was threatened or implied during the conversion process; some communities agreed to conversion but others were forced to convert)
(3) High (most communities were forced to convert)

(?) Missing data

v86. Was a foreign religion adopted through a top-down (those high in the social hierarchy converted first, followed by the general population) or a bottom-up (the general population converted first, followed by those high in the social hierarchy) process?

(1) Primarily a top-down process.
(2) Primarily a top-down process, although inroads had already been made with the general population.
(3) Mixed / Neither (e.g. Both high-status individuals and the general population converted at the same time, or the religion was adopted through a mixture of top-down and bottom-up processes.)
(4) Primarily a bottom-up process, although those in power showed little or no reluctance.
(5) Primarily a bottom-up process.

(?) Missing data

v89. Syncretic religious movements

A syncretic religious movement, for the purposes of coding, is an organised religious group that integrates features of an indigenous religion and one or more world religions.

(0) Absent
(1) Present, but did not survive to the present-day
(2) Present, and survived to the present day

(?) Missing Data

Section 3: Current Time Focus

v90. Current Time Focus

The current state coding sheet should describe the culture as it is at or around the time of coding. The time focus, then, should be as recent and narrow as is practical. This can be stated in the form of a calendar year or an interval, e.g. (2000-2010).

[Year]

(?) Missing data

v91. World Religions

What proportion of the indigenous population follows a world religion?

(0) Absent or minimal (Less than 5% of the indigenous population follows a world religion.)

(1) Present but minor (Between 5% and 25% of the indigenous population follows a world religion.)
(2) Present and major (Between 25% and 75% of the indigenous population follows a world religion.)
(3) Present and predominant, indigenous religion still practiced (Over 75% of the indigenous population follows a world religion, but the indigenous religion is still practiced by some members of the culture.)
(4) Present and predominant, indigenous religion no longer practiced (Over 75% of the indigenous population follows a world religion, and the indigenous religion is no longer practiced.)

(?) Missing data

v92. Dominant world religion

(1) Christianity
(2) Islam
(3) Hinduism / Buddhism
(4) Other

(?) Missing data

v93. Religious Syncretism – Institutional

Syncretism, for our purposes, is the fusion of features from the indigenous religion of the culture with features from one or more world religions. Institutional syncretism is syncretic belief and / or practice that occurs within or is explicitly endorsed by a religious institution, e.g. a church. Only syncretic beliefs and / or practices that survived to the present day or recent past should be considered.

(1) Low (The indigenous and world religion (s) have borrowed little from one another.)
(2) Medium (The indigenous and world religion(s) have remained distinct, but one or both has borrowed substantially from the other.)
(3) High (A new religion has formed which incorporates concepts and / or practices from both the indigenous and foreign religions.)

(?) Missing data

v94. Syncretism – Unofficial

Syncretism, for our purposes, is the fusion of features from the indigenous religion of the culture with features from one or more world religions. Unofficial syncretism is syncretic belief and / or practice that occurs outside of a religious institution and is not explicitly endorsed by that institution. Only syncretic beliefs and / or practices that survived to the present day or recent past should be considered.

(1) Low (Most people draw a clear distinction between the indigenous religion and the world religion (s), and do not combine indigenous and non-indigenous religious elements.)
(2) Medium (Most people draw a clear distinction between the indigenous religion and the world religion (s), but combine indigenous and non-indigenous religious elements.)
(3) High: (Most people do not draw a clear distinction between the indigenous religion the world religion, and combine indigenous and non-indigenous religious elements.)

(?) Missing data
