# Supplementary material for: Pulotu: Database of Austronesian Supernatural Beliefs and Practices
Source: PLoS One. 2015 Sep 23;10(9):e0136783. doi: 10.1371/journal.pone.0136783 (PMC4580586; doi:10.1371/journal.pone.0136783)
Supplement: S2 File — This file contains additional information of the methods used in the headhunting example. (DOCX) [file pone.0136783.s003.docx]

**S1 File. Supporting Methods for Headhunting Example.**

The coding criteria for the headhunting variable can be found in S1 File. We use Gray, Drummond and Greenhill (1) trees of Austronesian languages, pruning down the original 400 languages to the 88 spoken by Austronesian cultures for which headhunting data is available. Evidence of headhunting was found in 34 of the cultures sampled. First we tested for a phylogenetic signal in the distribution of Austronesian headhunting using Fritz and Purvis’ *D* (2) in the R package Caper (3). Our results indicate that the distribution of headhunting in Austronesian cultures is highly phylogenetically patterned (*D* = -.98), which can be seen in Fig. 5a. This result suggests that the practice of headhunting is highly dependent on cultural ancestry - cultures whose recent ancestors practiced headhunting are highly likely to practice headhunting themselves. As such, phylogenetic methods are required to model the origins and distribution of headhunting in Austronesia.

We then performed an ancestral state reconstruction using the software package *Bayestraits* (4) to model the history of headhunting in Austronesia across a sample of the 4199 most likely trees constructed from basic vocabulary items by Gray et al.’s (1). Using a sample of trees, rather than assuming one true tree, enables us to take into account phylogenetic uncertainty in the placement of language subgroups and timing of language divergences. To inform the priors for the MCMC analyses, we began by performing a Maximum Likelihood analysis for each tree in the sample. Across the sample the mean rate of gain (q01) was 0.0035, and the mean rate of loss (q10) was 0.0092. Using an MCMC method enables models of evolution to be tests across a sample of trees, and so does not assume one true tree. For the MCMC analysis we used a hyper-prior sampling from an exponential distribution with the range 0 to 0.02. Each analysis was run for 100,000,000 iterations, with the first 10,000,000 iteration excluded as a burn-in period. We replicated each model 3 times to ensure consistency, and all runs converged on highly similar values (S1 Table).

To provide a visual summary of our ancestral state reconstruction we created a maximum clade credibility tree using the program *TreeAnnotator* (5). Using the addnodes function in *Bayestraits* (6) we reconstructed each of the internal nodes of the consensus tree. The tree figure was then created using the R package Ape (7), with final editing in Adobe Illustrator and Photoshop. The resulting image is presented as Fig. 5a. At each node of the tree a pie chart represents the inferred state of that culture. Grey represents the proportion of the trees in the sample in for which that node is not present.

**S1 Table.** Summary of three independent ancestral state reconstructions of headhunting. The *q01* column indicates the rate at which cultures are inferred to gain headhunting, and *q10* indicates the rate at which cultures loose headhunting. Root p (0) indicates the probability that the culture at the root of the tree (proto-Austronesia) lacked headhunting, while Root p (1) indicates the probability that headhunting was present. Values for each run represent the mean from the posterior distribution.

| **Run** | ***q01*** | ***q10*** | **Root p (0)** | **Root p (1)** |
| --- | --- | --- | --- | --- |
| **1** | 0.0047 | 0.0095 | 0.01 | 0.99 |
| **2** | 0.0048 | 0.0095 | 0.01 | 0.99 |
| **3** | 0.0048 | 0.0094 | 0.02 | 0.98 |
| **Mean** | 0.0048 | 0.0095 | 0.01 | 0.99 |

**Supporting References**

1. Gray RD, Drummond AJ, Greenhill SJ. Language phylogenies reveal expansion pulses and pauses in Pacific settlement. Science. 2009;323(5913):479–83.

2. Fritz S, Purvis A. Selectivity in mammalian extinction risk and threat types: a new measure of phylogenetic signal strength in binary traits. Conserv Biol. 2010; 24(4):1042–51.

3. Orme D, Freckleton R, Thomas G, Fritz SA, Isaac N, Pea W. Caper: Comparative Analyses of Phylogenetics and Evolution in R. 2013.

4. Pagel M, Meade A. Bayesian analysis of correlated evolution of discrete characters by reversible‐jump Markov chain Monte Carlo. Am Nat. 2006;167(6):808–25.

5. Drummond AJ, Suchard MA, Xie D, Rambaut A. Bayesian phylogenetics with BEAUti and the BEAST 1.7. Mol Biol Evol. 2012;29:1969–73.

6. Pagel M, Meade A. Bayesian Analysis of Correlated Evolution of Discrete Characters by Reversible-Jump Markov Chain Monte Carlo. Am Nat. 2006;167(6):808–25.

7. Paradis E, Claude J, Strimmer K. APE: analyses of phylogenetics and evolution in R language. Bioinformatics. 2004;20(2):289–90.
